# The *C. elegans* TRPV channel proteins OSM-9 and OCR-2 contribute to aversive chemical sensitivity

**DOI:** 10.17912/micropub.biology.000277

**Published:** 2020-07-08

**Authors:** Emily A. Mehle, Savannah E. Sojka, Medha K.C., Rosy M. Zel, Sebastian J. Reese, Denise M. Ferkey

**Affiliations:** 1 Department of Biological Sciences, University at Buffalo, The State University of New York, Buffalo, NY 14260

**Figure 1:**
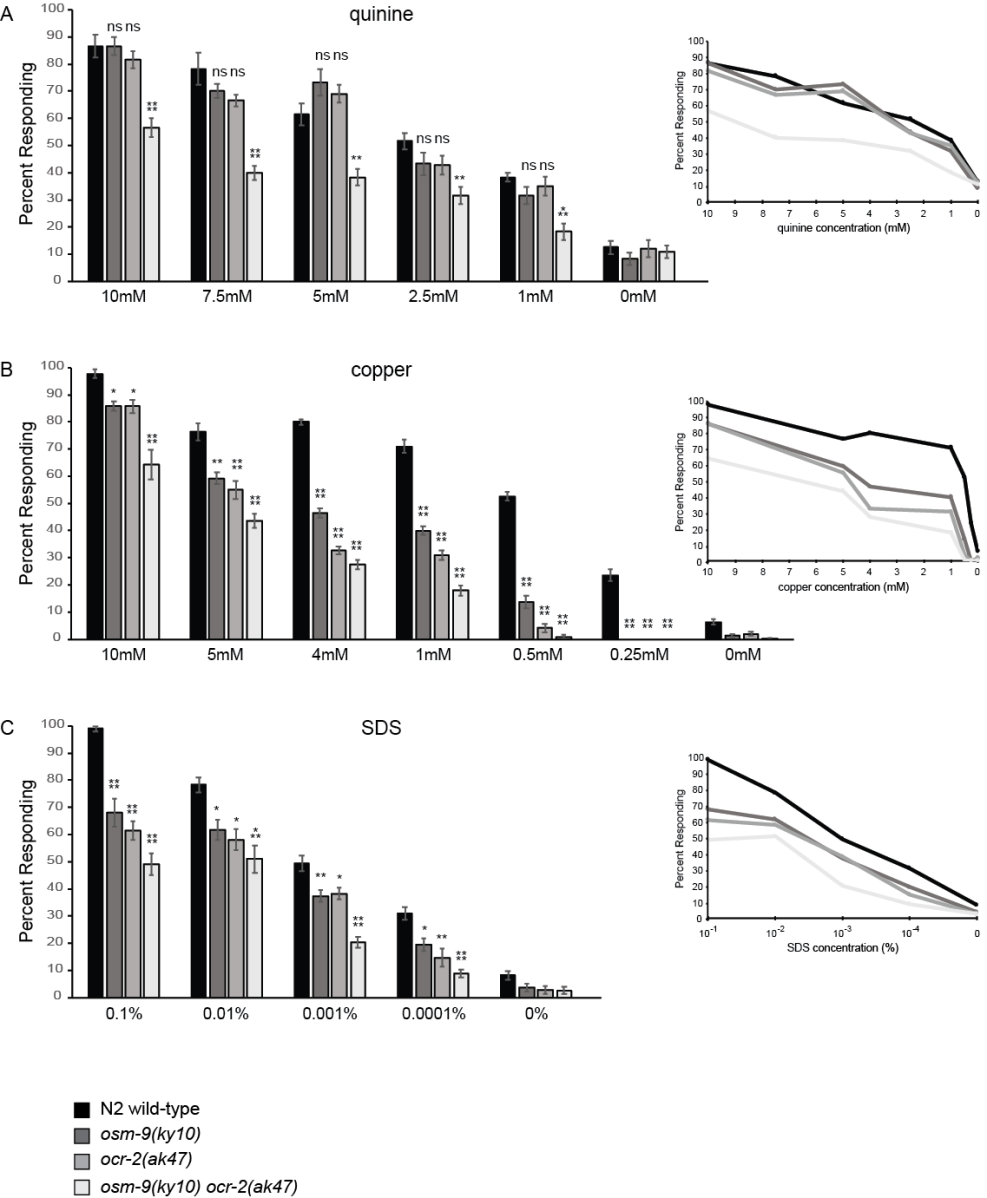
Behavioral response of well-fed young adults to aversive chemical stimuli was assessed using the drop assay 20 minutes after transfer to NGM plates lacking bacteria (“off food”). Bar graphs show the percentage of animals responding to the indicated concentrations of (A) the bitter tastant quinine, (B) the heavy metal copper (CuCl_2_), and (C) the detergent SDS. Inserts represent the corresponding dose-response curves for each genotype across the range of concentrations tested, for readability showing only the means from the bar graphs in panels A-C. n >/= 60 animals for each genotype were assayed at each concentration. Assays were performed on at least three separate days. All tastants were dissolved in M13 buffer, pH 7.4 (Wood 1988). Error bars represent the standard error of the mean (SEM). The one-way ANOVA with Tukey’s Honestly Significant Difference (HSD) was used for statistical analysis. * denotes p < 0.05, ** denotes p < 0.01, *** denotes p < 0.001, and **** denotes p < 0.0001 compared to wild-type animals. ns denotes p >/= 0.05. F values for 10mM – 1mM quinine, respectively: 16.7, 20.4, 16.1, 5.6, 9.2. F values for 10mM – 0.25mM copper, respectively: 19.9, 22.6, 245.7, 133.8, 212.5, 112.7. F values for 0.1% – 0.0001% SDS, respectively: 29.9, 7.6, 24.0, 11.5.

## Description

Transient receptor potential (TRP) channels are a family of cation channels that are important for response to diverse external stimuli across eukaryotes (Venkatachalam and Montell 2007; Samanta *et al.* 2018). In *C. elegans*, only members of the TRPV family, which includes *osm-9*, *ocr-1*, *ocr-2*, *ocr-3* and *ocr-4* (Colbert and Bargmann 1995; Colbert *et al.* 1997; Tobin *et al.* 2002), have been shown to play a role in chemosensory behavior (Bargmann 2006). OSM-9 and OCR-2 are co-expressed in six pairs of sensory neurons: AWA, ASH, ADL, ADF, PHA and PHB (Colbert *et al.* 1997; Tobin *et al.* 2002). In these cells, they are thought to come together to function as a single channel complex to mediate sensory transduction since their localization to the cilia is mutually dependent upon each other (Tobin *et al.* 2002).

In the literature, OSM-9 and OCR-2 are often referred to as being required for all ASH-mediated avoidance behaviors. Indeed, *osm-9* and *ocr-2* mutant animals are defective for response to nose touch and high osmolarity, although *ocr-2* mutants did retain some ability to respond to the highest osmotic strength tested (4M) (Colbert *et al.* 1997; Tobin *et al.* 2002). *osm-9* and *ocr-2* mutants are also severely defective in avoidance of 2-octanone (Tobin *et al.* 2002) and 1-octanol (Ezak *et al.* 2010). Both mutants are defective in avoidance of high pH (Sassa *et al.* 2013; Wang *et al.* 2016), and *osm-9* mutants are defective in CuSO_4 _avoidance when the assay was performed in a plate chemotaxis format (Wang *et al.* 2015). However, *osm-9* and *ocr-2* single mutants, as well as *osm-9 ocr-2* double mutant animals, retain partial response to bitter tastants, including quinine, when tested at 10mM (Hilliard *et al.* 2004; Ezak *et al.* 2010). Animals lacking either or both channels also retain an intermediate level of response to the heavy metal copper (10mM CuCl_2_) and the detergent SDS (0.1%) (Ezak *et al.* 2010).

We sought to determine the extent to which OSM-9 and OCR-2 contribute to the ASH-mediated behavioral avoidance of quinine (Hilliard *et al.* 2004), copper (Sambongi *et al.* 1999) and SDS (Hilliard *et al.* 2002). We used the drop assay as previously described (Hilliard *et al.* 2002; Fukuto *et al.* 2004; Hilliard *et al.* 2004; Ezak *et al.* 2010; Krzyzanowski *et al.* 2013) to assess the percentage of wild-type, *osm-9(ky10)*, *ocr-2(ak47)*, and *osm-9(ky10) ocr-2(ak47)* animals responding to each soluble chemical stimulus across a range of concentrations (Figure 1).

Although in one study *osm-9* mutant animals were reported to have a modest but statistically significant defect in 10mM quinine avoidance (Hilliard *et al.* 2004), we found that individual loss of neither OSM-9 nor OCR-2 function affected response to 10mM quinine (Figure 1A), similar to our previous report (Ezak *et al.* 2010). Further, *osm-9* and *ocr-2* single mutants responded similarly to wild-type animals across the range of quinine concentrations tested (10mM – 1mM, Figure 1A). Only in the *osm-9 ocr-2* double mutant animals was a partial defect in quinine avoidance seen, at each concentration (Figure 1A).

In response to copper (CuCl_2_), at each concentration the *osm-9* and *ocr-2* single mutants, as well as the *osm-9 ocr-2* double mutant, were partially defective in the avoidance response (Figure 1B). Only at the lowest concentrations tested (0.5mM and 0.25mM) were either the single or double mutant animals essentially lacking response (Figure 1B). Similarly, in response to SDS only a partial avoidance defect was seen for *osm-9* and *ocr-2* single mutants at 0.1% – 0.001%, with the double mutant showing a somewhat greater defect at 0.001% (Figure 1C).

Taken together, assaying animals at intermediate concentrations of quinine, copper and SDS demonstrated that even when lacking the function of both OSM-9 and OCR-2, a substantial percentage of animals retained behavioral response to these aversive stimuli. Thus, although OSM-9 and OCR-2 are key components in ASH-mediated chemosensory avoidance, their loss leads to diminished, but not absent, avoidance responses at most concentrations tested. This suggests that an additional channel(s) may contribute to chemical avoidance in the absence of these TRPV channels.

## Reagents

The strains N2 Bristol wild-type, CX10 *osm-9(ky10)* and CX4544 *ocr-2(ak47)* were obtained from the *Caenorhabditis* Genetics Center, which is funded in part by the National Institutes of Health – Office of Research Infrastructure Programs. LX748 *osm-9(ky10) ocr-2(ak47)* was a gift from Michael Koelle and has not been sent to the CGC.
